# Supplementary material for: Full-Length Model of SaCas9-sgRNA-DNA Complex in Cleavage State
Source: Int J Mol Sci. 2023 Jan 7;24(2):1204. doi: 10.3390/ijms24021204 (PMC9867433; doi:10.3390/ijms24021204)
Supplement: Supplementary file 1 [file ijms-24-01204-s001.zip › ijms-2084294-supplementary.pdf]

International Journal of *Molecular Sciences*

MDPI

Supplementary Materials

Full-Length Model of SaCas9-sgRNA-DNA Complex in Cleavage State

Wenhao Du [1], Haixia Zhu [1], Jiaqiang Qian [1], Dongmei Xue [2], Sen Zheng [2] and Qiang Huang [1,2,*]

[1] State Key Laboratory of Genetic Engineering, Shanghai Engineering Research Center of Industrial Microorganisms, MOE Engineering Research Center of Gene Technology, School of Life Sciences, Fudan University, Shanghai 200438, China

[2] Multiscale Research Institute for Complex Systems, Fudan University, Shanghai 201203, China

* Correspondence: huangqiang@fudan.edu.cn

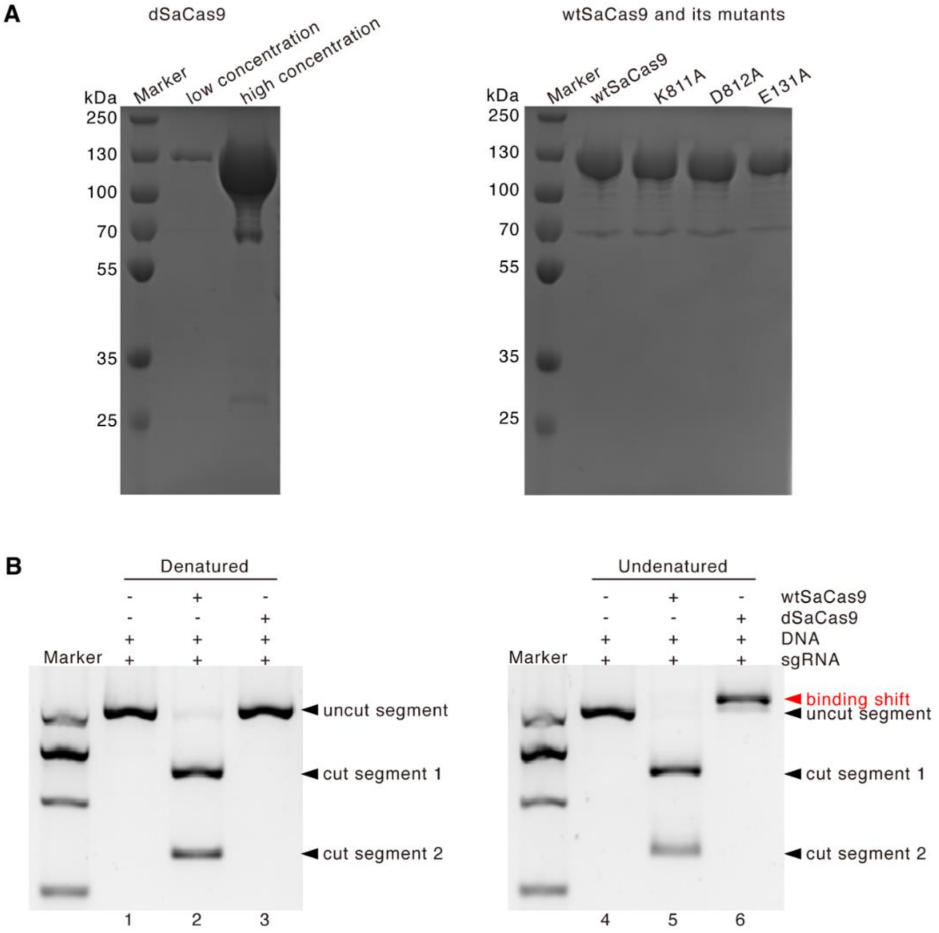

Figure S1. Detection of purified SaCas9 and its mutants. (**A**) SDS-PAGE results for the purified SaCas9 and its mutants. (**B**) Detection of the wtSaCas9/dSaCas9-sgRNA-DNA ternary complex by agarose gel electrophoresis.

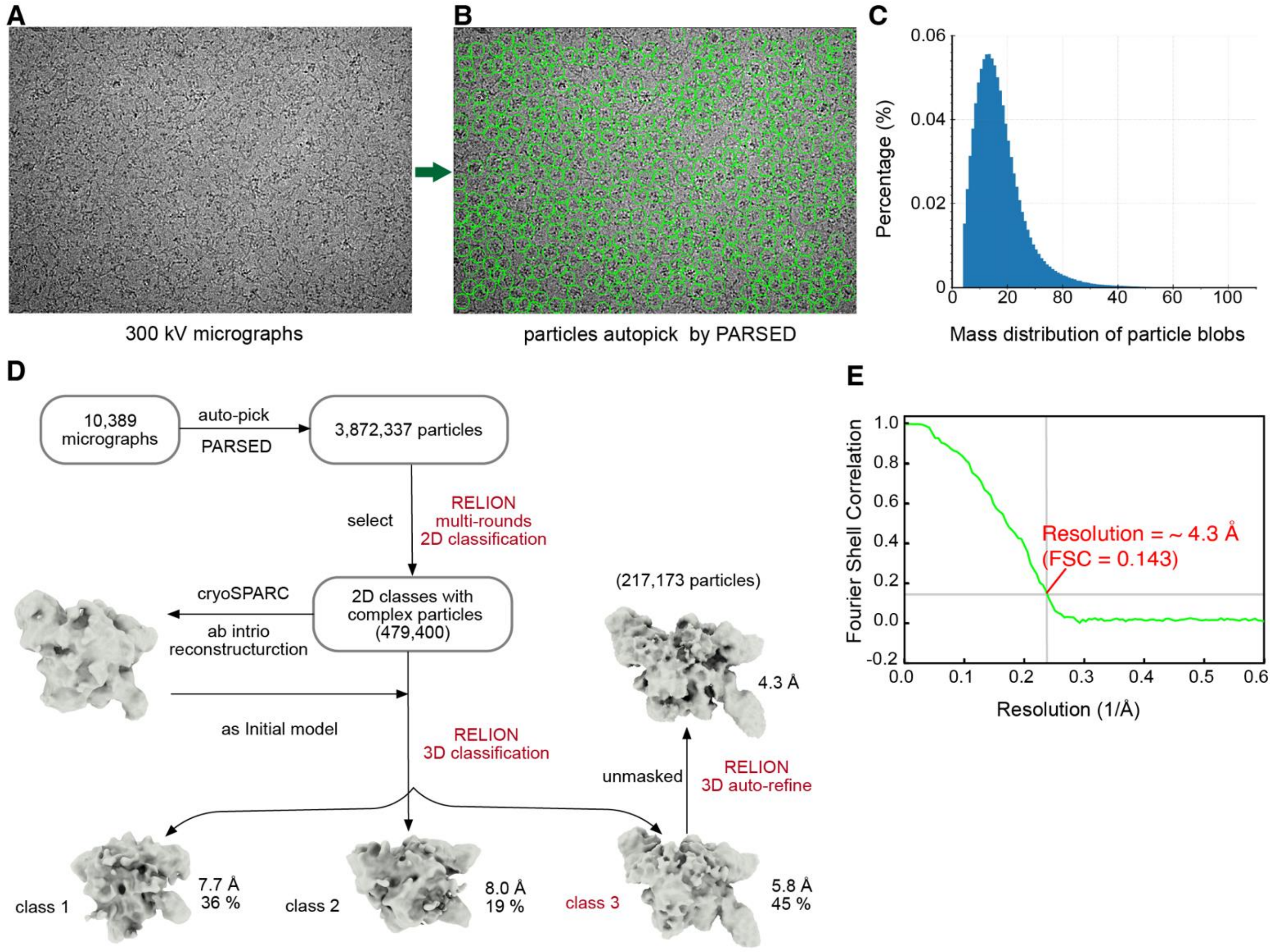

Figure S2. The single-particle 3D reconstruction of the SaCas9-sgRNA-DNA ternary complex. (**A**) A typical raw micrograph of the SaCas9-sgRNA-DNA ternary complex. (**B**) Particle picking of the cryo-EM micrographs with the program PARSED. Corresponding picked particles indicated by the green circles. (**C**) The mass distributions of the particle blobs calculated by PARSED. Only one sharp peak exists in the dataset, so all the picked particles were selected for the single-particle reconstruction. (**D**) Workflow for the single-particle 3D reconstruction of the SaCas9-sgRNA-DNA ternary complex. (**E**) FSC curves for the cryo-EM density maps.

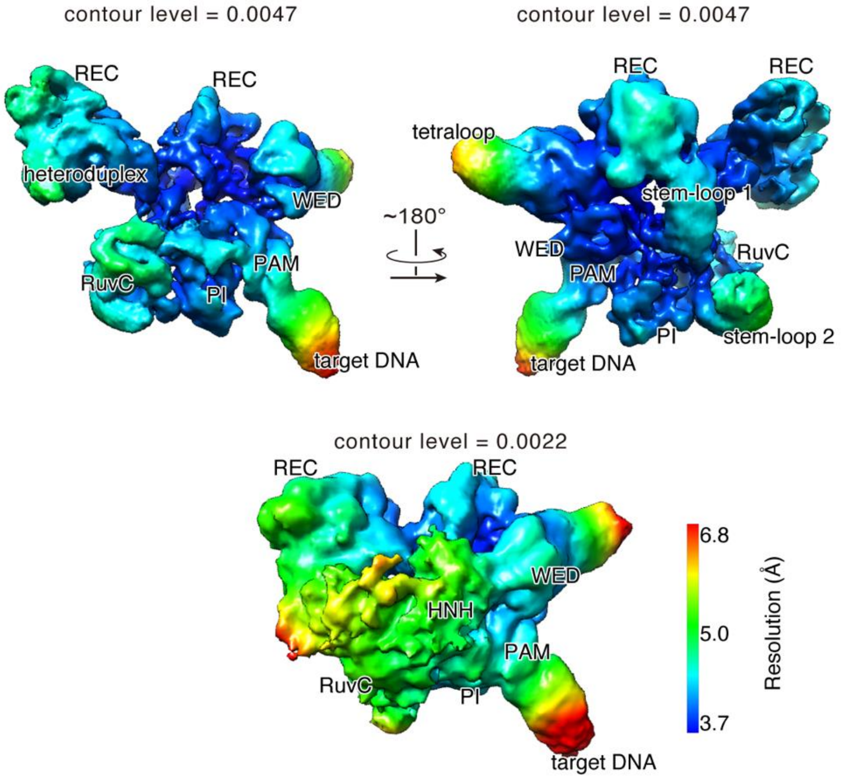

Figure S3. The local resolution of the cryo-EM density map. The resolutions from high to low (3.7 ~ 6.8 Å) are indicated by the blue to red gradient color band.

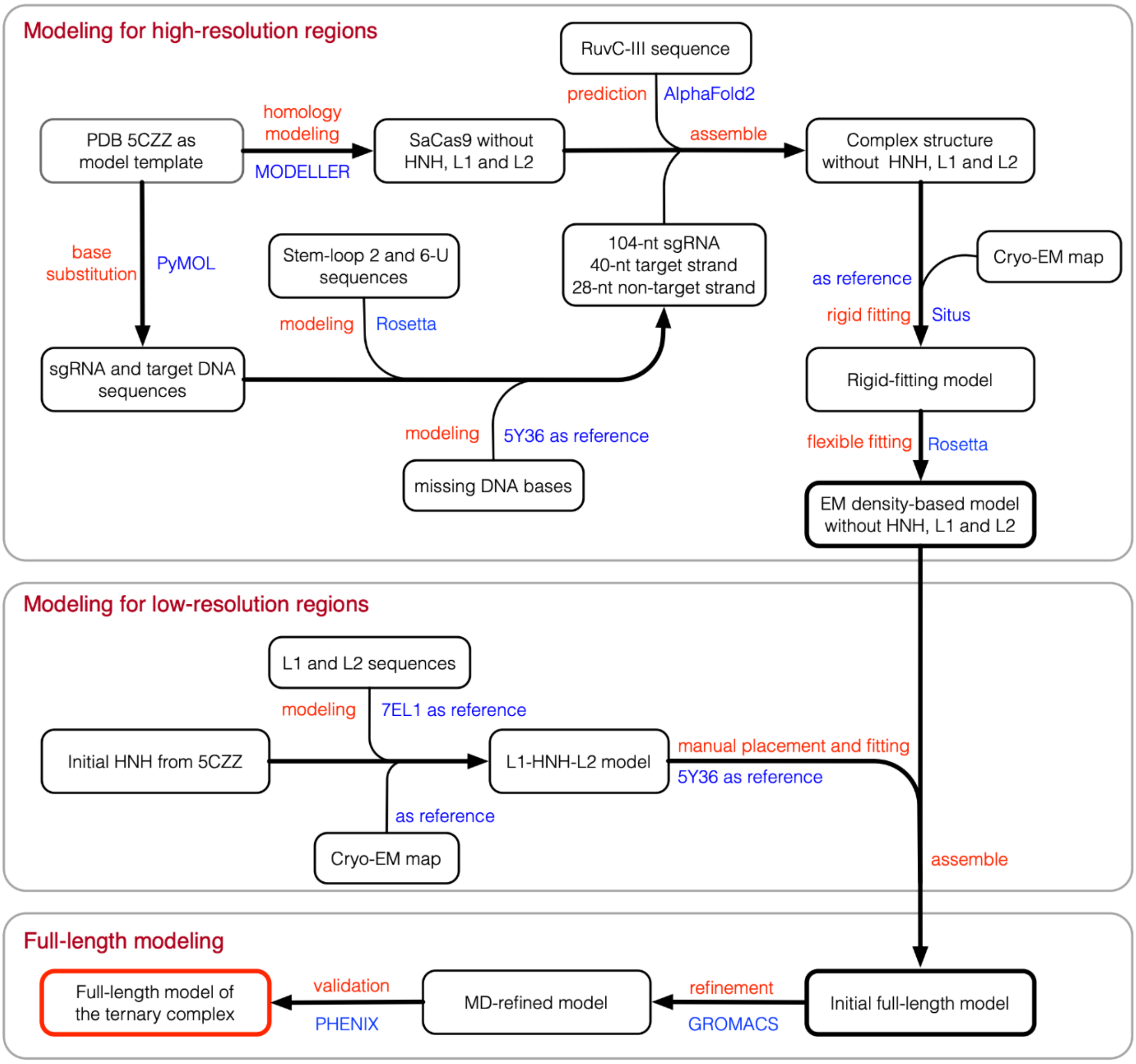

Figure S4. Flowchart for building the full-length model of the SaCas9-sgRNA-DNA complex.

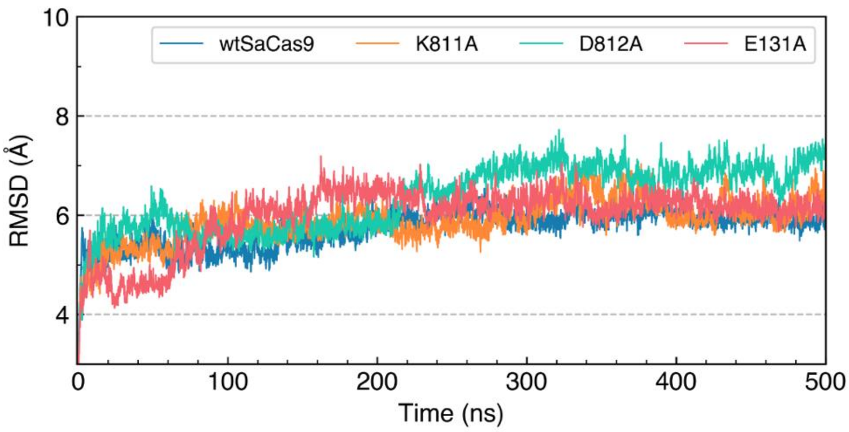

Figure S5. RMSDs of SaCas9 and its mutants during the MD simulations with respect to the initial MD structures.

Table S1. Primer sequences used in this study.

Names	Sequences(5′→3′)
Construction of the SaCas9 mutants	
dSaCas9-10A_F	AACTACATCCTGGGCCTGGCCATCGGCATCACCAGCGT
dSaCas9-10A_R	ACGCTGGTGATGCCGATGGCCAGGCCCAGGATGTAGTT
dSaCas9-580A_F	AAGGTGCTCGTGAAGCAGGAAGAAGCCAGCAAGAAGGGCAACCGGACC
dSaCas9-580A_R	GTCCGGTTGCCCTTCTTGCTGGCTTCTTCCTGCTTCACGAGCACCTTG
SaCas9-131A_F	CTGCTCTTTGGTGGACAGTGCGTTGCCGGTGTCCTCTT
SaCas9-131A_R	AAGAGGACACCGGCAACGCACTGTCCACCAAAGAGCAG
SaCas9-811A_F	CTGAACGGCCTGTACGACGCCGACAATGACAAGCTGAAA
SaCas9-811A_R	TTTCAGCTTGTCATTGTCGGCGTCGTACAGGCCGTTCAG
SaCas9-812A_F	GAACGGCCTGTACGACAAGGCCAATGACAAGCTGAAAAAGC
SaCas9-812A_R	GCTTTTTCAGCTTGTCATTGGCCTTGTCGTACAGGCCGTTC
Amplification of sgRNA	
sgRNA_F	TAAGTTGGGTAACGCCAGGGTTTTC
sgRNA_R	AAAAAAATCTCGCCAACAAGTTGACG
Amplification of substrate DNA	
DNA_F	CGGAAATGTTGAATACTCATACTCT
DNA_R	GAGTCAGTGAGCGAGGAAGCGGAAG

Table S2. Cryo-EM data collection, processing, refinement and validation statistics.

Sample	SaCas9-sgRNA-DNA complex (EMD-32104, PDB ID: 7VW3)
**Data collection**	
Electron microscope	Titan Krios G3i
Camera	Gatan K3 Bioquantum direct electron detector
Pixel size (Å/pix)	0.85
Defocus range (μm)	−1.8 to −2.6
Exposure time (second)	7.6
Total dose (e/Å^2)	38
Movie frames (no.)	38
Total micrographs (no.)	10,389
**Reconstruction**	
Software	RELION
Particles for 2D classification	3,872,337
Particles for 3D classification	479,400
Particles in the final map (no.)	217,173
Symmetry	C1
Final resolution (Å)	4.3
FSC threshold	0.143
**Model building**	
Software	Rosetta
**Refinement**	
Software	GROMACS, PHENIX
**Model composition**	
Protein	1052
Nucleotide	192
**Validation**	
Clash score	23
**R.m.s. deviations**	
Bond lengths (Å)	0.41
Bond angles (°)	0.73
**Ramachandran plot**	
Favored (%)	90
Allowed (%)	9
Outliers (%)	1
